# Circadian rhythmicity in emerging mood disorders: state or trait marker?

**DOI:** 10.1186/s40345-015-0043-z

**Published:** 2016-01-13

**Authors:** Ashlee B. Grierson, Ian B. Hickie, Sharon L. Naismith, Daniel F. Hermens, Elizabeth M. Scott, Jan Scott

**Affiliations:** Brain and Mind Centre, The University of Sydney, Sydney, Australia; Charles Perkins Centre, Brain and Mind Centre, The University of Sydney, Sydney, Australia; Notre Dame University, Sydney, Australia; Sydney Medical School and Brain and Mind Centre, The University of Sydney, Sydney, Australia; Academic Psychiatry, Institute of Neuroscience, Newcastle University, Newcastle, UK

**Keywords:** Circadian rhythm, Bipolar, Unipolar, Adolescence, Young adult, Trait

## Abstract

**Background:**

Circadian rhythm disturbances overlap with the symptoms of mood episodes and may trigger or prolong mood symptoms. There is limited research on the role of circadian disturbances in mood disorders in young people and/or first episode cases of unipolar and bipolar disorders.

**Methods:**

Actigraphy was undertaken for about 14 days in 63 post-pubertal individuals aged 13–25 years with a recent onset of a mood disorder meeting recognised diagnostic criteria. We examined associations between three easily interpretable markers of circadian rhythm activity (amplitude, acrophase and rhythmicity index) and demography and clinical characteristics. Then, circadian markers were compared between diagnostic groups, controlling for potential confounders.

**Results:**

Longer duration of illness was correlated with reduced circadian rhythmicity and lower levels of activity over 24 h. A delay in the timing of maximum activity was associated with the level of manic but not depressive symptoms. The circadian rhythmicity index differentiated unipolar from bipolar cases, and in bipolar but not unipolar disorder, the rhythmicity was less robust in those with more severe manic or depressive symptoms.

**Conclusions:**

Less robust circadian rhythmicity, especially associated with increasing symptom severity, may represent a more specific or a trait marker of young people with mood disorders who are at higher risk of a bipolar course of illness.

## Background

Circadian rhythm disturbances are of considerable interest to mood disorder researchers as many of the phenomena that accompany disruptions in the sleep–wake cycle overlap with the symptoms of mood episodes or may trigger or prolong mood symptoms (e.g. McClung 2013; Robillard et al. 2013a). However, most investigations of circadian rhythm disruptions in mood disorders are focused on older adult samples with established unipolar (UP) or bipolar disorders (BD). Often these individuals have a history of multiple mood episodes, accompanied by a high prevalence of mental and physical comorbidities, complex treatment regimens and/or elevated body mass index (BMI) (Geoffroy et al. 2015). As such, it is difficult to disentangle which abnormalities represent causes and which are consequences of mood episodes (Bellivier et al. 2015). There is limited research on the role of circadian disturbances in UP and BD in young people, especially related to first episode onset or the first treated episode (e.g. Ritter et al. 2011; Castro et al. 2015; Ng et al. 2015).

One of the problems in making comparisons between studies undertaken in young people with emerging mood disorders is that the sampling strategies and the parameters measured vary considerably between studies. For example, some studies focus on individuals at high risk of developing psychotic or mood disorders (identified by the presence of attenuated symptoms or temperamental style), whilst others recruit mixed samples of symptomatic and asymptomatic offspring of parents with UP or BD (e.g. Jones et al. 2006; Adan et al. 2010; Hidalgo et al. 2009; Castro et al. 2015; Park et al. 2015). Most of these studies use self-report assessments of sleep patterns or self-ratings of circadian rhythm such as the Morning–Eveningness Questionnaire (MEQ) (Horne and Ostberg 1976); the studies that do use objective measures such as actigraphy often focus on more traditional measures of sleep, such as sleep latency, waking after sleep onset and total sleep time (e.g. Castro et al. 2015). Studies measuring circadian parameters and using actigraphy have often recruited young people with a wide range of disorders (anxiety, UP, BD, psychosis), but failed to take into account in the analyses the other variables known to influence circadian rhythms, such as age, pubertal status or BMI, etc., (Adan et al. 2010; Mansour et al. 2005; Caci et al. 2009; Kim et al. 2010; Tranel et al. 2015).

Despite the methodological heterogeneity (noted above), there is a growing research literature on actigraphic measures of circadian rhythms in youth. However, the clinical translation of some of these studies is reduced because many clinicians are unfamiliar with the relevance of some measures and/or are uncertain how to interpret some of the circadian parameters (e.g. alpha, beta, mesor) (Bellivier et al. 2015). As such, it can be helpful to select circadian markers that have shown differences between young people with mood disorders (compared to healthy control or other cases with mental disorders), and that can be easily understood by clinicians. The three obvious circadian parameters are amplitude, acrophase and circadian rhythmicity index. Several studies report lower 24-hour activity rhythms (amplitude) or changes in dim light melatonin onset for UP and BD (Teicher et al. 1993; Armitage et al. 2004; Robillard et al. 2013b; 2015), and for individuals at risk of BD (Bullock and Murray 2014; Castro et al. 2015). The timing of maximal activity within a 24-h period (acrophase) has shown mixed results in UP and BD (Teicher et al. 1993; Robillard et al. 2013a; 2015), but there is emerging evidence of lower robustness of circadian rhythmicity in broad populations of young patients compared to healthy controls (e.g. Carpenter et al. 2015). The latter could be an interesting avenue for youth research as Gonzalez and colleagues (2014) recently demonstrated that a lower circadian rhythmicity index was a useful marker of illness severity in adults with BD and was correlated with total manic symptom score and with some individual manic symptoms. However, rhythmicity in comparable populations of UP and BD cases remains unexplored.

This study examines the role of selected markers of circadian rhythmicity in emerging mood disorders in young people aged 13–25 years who presented for the first time to clinical services. We focused on three key, easily interpretable parameters of circadian regulation, namely amplitude, acrophase and the circadian rhythmicity index. The aims were (a) to examine the associations between circadian parameters and age, gender, BMI, duration of illness and symptom severity in the total sample; (b) to compare these circadian parameters in BD and UP (after controlling for potential confounders).

## Methods

### Participants

With the approval of the Human Research Ethics Committee of The University of Sydney, individuals attending the local youth mental health services in and around Sydney, Australia (Youth Mental Health Clinic at the Brain & Mind Research Institute, at The University of Sydney, and headspace in Campbelltown) were invited to participate in a series of studies of mental and physical health, including research on sleep and actigraphy.

Individuals included in this study were aged 13–25 years, had a recent onset of a mood disorder meeting DSM-IV R criteria (APA. 2000), and were willing and able to give written informed consent to participate and to comply with study procedures. In those aged <16 years, additional written consent was obtained from their parents or legal guardians. The exclusion criteria were (i) clinically assessed IQ <70 or intellectual impairment, and/or history of head injury, (ii) mood disorder secondary to a medical condition or psychotic disorder, (iii) primary substance or alcohol misuse disorder, (iv) risk of suicide or self-harm, (v) regular use of medications that affect sleep, melatonin, circadian rhythms or alertness, (vi) evidence of other sleep (e.g. sleep apnoea, narcolepsy), neurological (e.g. epilepsy) or primary medical conditions that could explain the current depression and/or contribute to sleep–wake dysfunction, (vii) recent trans-meridian travel (i.e. potential for jet lag) or regular shift work.

### Assessments

As described previously, participants completed a detailed clinical assessment conducted with a structured clinical proforma (Hickie et al. 2014; Scott et al. 2014, 2015). For this study, the following data were used:

1. Demographics—current age and gender.
2. Clinical history—a research psychologist or psychiatrist established that the individual had a UP or BD that met DSM-IV R diagnostic criteria. Although the cases were presenting to clinical services for the first time with syndromal illness episodes, this was not necessarily the first experience of clinical symptoms or sub-threshold syndromes. As such, we included a measure of time since onset of any symptoms, which is referred to as the duration of illness (and was estimated as current age minus age at first onset of any psychiatric symptoms) (Scott et al. 2014). Basic details of current medications (class of medications prescribed) and body mass index (BMI) were recorded.
3. Symptom severity—observer ratings of mood symptoms were undertaken using the 17-item version of the Hamilton Rating Scale for Depression (HRSD; Hamilton, 1960) and the 11-item version of the Young Mania Rating Scale (YMRS) (Young et al. 1978) in all participants.
4. Sleep profile—self-report and observer-rated assessments of sleep were undertaken for other studies by the research group, but are not reported here (for details see Robillard et al. 2014).

In this study, objective recordings of sleep profile were undertaken for between 5 and 14 days (median 10 days) using an actiwatch (Actiwatch-64/L/2/Spectrum, Philips Respironics, USA) and sleep–wake detection was conducted automatically with Actiware 5.0 software (Philips Respironics) using the medium sensitivity threshold.

To characterise the circadian profile of the activity-rest cycle, individual actigraphy datasets were fitted to an extended Cosinor model using GraphPad Software. In this study, we estimated three key parameters, namely:

(i) Amplitude: a measure of the range of activity levels across the 24-h period,
(ii) Acrophase: a phase marker that can be used as a measure of an advance or delay in time of maximum activity, and
(iii) Circadian Rhythmicity Index (R2): an indicator of the strength or robustness of circadian rhythms, and also referred to as R2 or the coefficient of determination. The circadian rhythmicity index is a ‘goodness of fit’ measure and higher values indicate smaller discrepancies between actigraphy data and values predicted by the Cosinor model and higher scores represent more robust rhythms (for an explanation of how to undertake the analysis see Marler et al. 2006).

### Statistical analyses

All analyses used an alpha of 0.05 and were conducted using SPSS (version 21).

Clinical and demographic characteristics for the UP and BD groups were compared using univariate analyses (*t*-tests, Mann–Whitney *U* and Fisher’s exact test). Pearson’s product moment correlational analyses were used to examine any associations between each circadian parameter (amplitude, acrophase and R2) and continuous variables measuring demographic and illness characteristics i.e. current age, duration of illness, BMI, HRSD and YMRS in the total sample. Next, we conducted an analysis of variance (ANOVA) for each circadian parameter according to mood disorder subtype (UP or BD) with gender, current age, duration of illness, BMI, HRSD and YMRS as covariates.

## Results

As shown in Table 1, the groups were well matched for current age and gender and there were no statistically significant differences in duration of illness, BMI or symptom severity scores. The YMRS scores were not normally distributed, but the higher median score in the BD group was not significantly greater than in the UP group. Most cases of both UP and BP were being prescribed only one medication, and the majority of the sample received an antidepressant. Some BD cases were co-prescribed a mood stabilizer or atypical antipsychotic, but prescribing patterns did not differ significantly between the diagnostic groups.

**Table 1:** Sample characteristics

	Unipolar disorder (*N* = 33)	Bipolar disorder (*N* = 30)
	Mean (SD)	Mean (SD)
Current age^a^	18.66 (3.01)	19.95 (2.13)
Duration of illness	4.45 (3.59)	5.92 (2.87)
Body Mass Index	23.67 (2.89)	24.05 (4.77)
Hamilton Rating Scale for Depression	13.97 (7.90)	12.44 (7.08)
Young Mania Rating Scale^b^ Median (IQR)	2 (0, 3)	3.5 (1, 5)
	Number (%)	Number (%)
Females	20 (61)	19 (63)
Medications^d^		
Antidepressant	25 (76)	19 (77)
Atypical antipsychotic	5 (15)	7 (23)
Mood stabilizer^c^	2 (6)	5 (17)
Other	2 (6)	0 (0)

*IQR* Interquartile range

^a^ *t*-test: *t* = 1.94, *p* = 0.06

^b^Mann–Whitney *U* test: *p* = 0.07

^c^Fisher’s exact test: *p* = 0.09

^d^Total for all medications exceeds 100 % as some individuals were prescribed >1 medication

For the total sample, the mean amplitude was 2.04 (SD 0.76), the mean acrophase was 16.19 h (SD 1.65) and the mean R2 was 0.42 (SD 0.13); there were no significant differences according to gender. As shown in Table 2, current age was significantly negatively correlated with R2 (*r* −0.40; *p* = 0.001), whilst duration of illness was significantly negatively correlated with amplitude (*r* −0.25; *p* = 0.045) and with R2 (*r* −0.33; *p* = 0.01). The BMI was not associated with any circadian parameters. With regard to current symptom severity, the HRSD was not significantly associated with circadian parameters, but the YMRS score was significantly positively correlated with acrophase (*r* 0.28; *p* = 0.03).

**Table 2:** Correlations between clinical characteristics and circadian parameters

	Current age	Duration of illness	Body mass index	HRSD	YMRS
Amplitude	0.06	−0.25*	0.10	−0.03	−0.10
Acrophase	0.21	0.12	0.03	0.07	0.28*
Circadian rhythmicity index (R2)	−0.40**	−0.33**	−0.12	−0.20	−0.17

*p < 0.05; **p < 0.01 (see text for exact levels of significance)

**Fig. 1:**
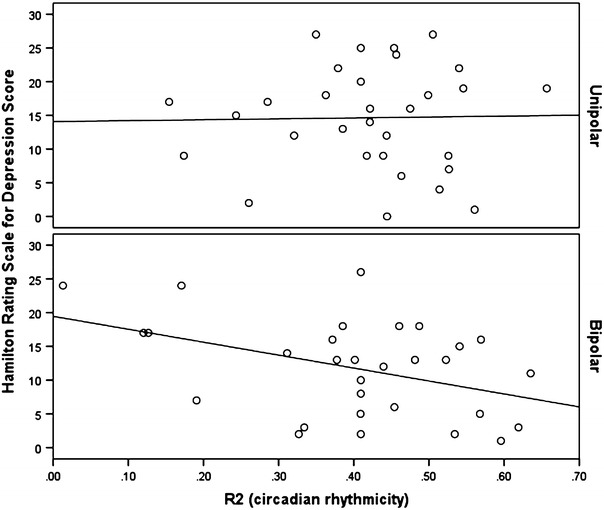
Correlation between Hamilton rating scale for depression score and circadian rhythmicity (R2) in unipolar and bipolar disorders

**Fig. 2:**
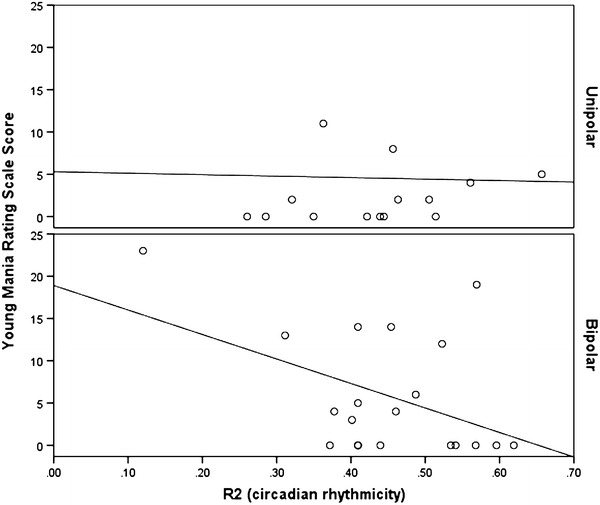
Correlation between Young mania rating scale score and circadian rhythmicity (R2) in unipolar and bipolar disorders

The ANOVA demonstrated that, after taking into account potential confounders (age, gender, duration of illness, BMI, HRSD, YMRS), differences between the diagnostic groups were significant for R2 (mean UP v. BD = 0.43 v 0.40; *F* = 2.34; df = 6, 56; *p* = 0.044), but not for amplitude (mean = 2.14 v. 1.84; *F* = 1.51; *p* = 0.09) or acrophase (mean = 16.07 v 16.30 h; *F* = 1.17; *p* = 0.33). As shown in Figs. 1 and 2, symptom severity and diagnostic group were the main to the significant model for R2. The regression slopes for the correlations between R2 and HRSD or R2 and YMRS are different in BD compared to UP cases. There is little variation in R2 across different levels of symptom severity in UP, but higher levels of either manic or depressive symptoms are associated with a significantly lower circadian rhythmicity index in BD cases.

## Discussion

The study set out to explore two aspects of circadian rhythms in youth with mood disorders. First, the associations between the selected circadian parameters and basic demographic and clinical characteristics (independent of mood disorder subtype), and second, whether any specific parameters were more likely to be disturbed in UP compared to BD.

The correlational analyses undertaken in this study show a relationship between the selected circadian rhythm parameters, the first treated episode of mood disorders in youth and current age. First, longer duration of illness (defined as the time since the first onset of any symptoms) was associated with a reduced circadian rhythmicity index and lower levels of activity over 24 h. Second, there was a significant inverse correlation between increasing age and robustness of circadian rhythmicity. Third, acrophase was associated with current mental state, being significantly correlated with the level of manic but not depressive symptoms.

Taken together, these findings could suggest that weakened rhythmicity and lower 24-h activity are markers of vulnerability to developing syndromal episodes of UP or BD. We cannot state categorically that they are trait markers for UP or BD as, even in this first-treated sample, many individuals had a history of subsyndromal symptoms. So, the circadian disturbances could be a consequence of longer duration of symptoms. However, we can hypothesis that these circadian parameters are indicators that mood symptoms will meet the diagnostic threshold for a full syndromal episode (Bullock and Murray 2014; Castro et al. 2015). In contrast, the acrophase findings suggest that the timing of maximum activity may be a state marker of manic symptom severity. This finding is in line with some but not all previous studies. Several studies in young people failed to show an association between acrophase and depression (Teicher et al. 1993; Robillard et al. 2015), whilst several studies in older adults have noted an association between mania and acrophase (although findings are inconsistent with regard to phase advance or delay) (Salvatore et al. 2008; Ng et al. 2015).

We suggest that the findings regarding current age are important as they emphasise that there is a developmental trajectory for the variability in 24-h rhythms that should be taken into account when planning studies of sleep and mood disorders (Mansour et al. 2005; Caci et al. 2009; Kim et al. 2010). This is especially important in studies that focus on populations of adolescents and young adults, as age-matching may be needed to avoid misinterpreting any circadian disruption as an association with illness characteristics as compared to demography (Fares et al. 2015). Similarly, previous studies have demonstrated an association between gender and/or increased BMI and circadian disturbance in adolescents and young adults (e.g. Arora and Taheri 2014; Tranel et al. 2015), and we suggest that these factors also need to be routinely reported in future studies. The lack of significant associations between gender and circadian disturbance may be due to recruiting a post-pubertal sample (or the sample size); but the lack of association between BMI and circadian disturbance was somewhat unexpected. It may be because the sample did not manifest high levels of obesity (the mean BMI was <25) or it may be a consequence of the relatively small sample size (Boudebesse et al. 2015).

The examination of mood disorder subtypes suggests that the circadian rhythmicity index demonstrates a stronger association with illness episodes in BD as compared to UP. After taking into account possible confounders, we found that rhythmicity in BD was lower than in UP. Furthermore, whilst there were little apparent differences in rhythmicity across all levels of symptom severity in UP, reduced rhythmicity was associated with both higher HRSD and higher YMRS scores in BD. A difference between UP and BD might be anticipated for R2 and YMRS scores, as we would not anticipate high levels of manic-like symptoms in the UP group and also, previous publications suggest that there is heightened variability in circadian markers across mood phases in BD (Novakova et al. 2015). However, the finding that the BD group, but not the UP group, also showed lower rhythmicity in association with higher levels of depressive symptoms is an important finding that warrants replication. The findings may indicate that weakened circadian rhythms, especially associated with increasing severity of depressive symptoms, is a more specific marker of BD than of UP in youth.

The current study has a number of limitations. First, the sample size limits the number of statistical analyses that could be planned and performed. Also, it meant we could not justify undertaking any further sub-group analyses to determine if any specific constellations of manic or depressive symptoms were associated with weakened rhythmicity (as reported by Gonzalez et al. 2014). Second, although the age range was limited to the post-pubertal and early adult period, there were trends towards significant differences between the UP and BD groups in mean age (*p* = 0.06) and the BD group had a longer mean duration of symptoms. Without specific age and gender matching, we cannot rule out the possibility that these differences contributed to some extent to the findings in this study. Third, although the cases were presenting to the clinical services for the first time, the study participants were nearly all receiving medication by the time they undertook the actigraphy recording of their sleep–wake patterns. Although there were no significant differences in medications received by the UP and BD cases, it is possible that some findings might be associated with, or the findings were enhanced by any effects associated with the medications prescribed. Lastly, although symptom levels were in the mild to moderate range, it does make it difficult to distinguish state and trait abnormalities in circadian rhythms.

## Conclusions

In conclusion, the study selected circadian markers (acrophase, amplitude and circadian rhythmicity) that are easy to interpret clinically and that capture a holistic picture of circadian rhythm disruption in mood disorders. The findings provide evidence for circadian disturbances in youth with mood disorders that warrant further investigation, especially the possibility that the circadian rhythmicity index is lower in BD compared to UP and is less robust in the face of increasing levels of manic or depressive symptoms.

## Abbreviations

ANOVA: analysis of variance
BMI: body mass index
BD: bipolar
DSM-IV-R: Diagnostic and Statistical Manual of Mental Disorders Fourth Edition Revised
HRSD: Hamilton Rating Scale for Depression
IQ: intelligence quotient
MEQ: morning–eveningness questionnaire
R2: circadian rhythmicity index
UP: unipolar
YMRS: Young mania rating scale
